# Non-covalent interactions in MOFs: a quantum approach to gas adsorption and molecular encapsulation

**DOI:** 10.3389/fchem.2025.1579977

**Published:** 2025-06-06

**Authors:** Erika Medel, Rubicelia Vargas

**Affiliations:** Departamento de Química, División de Ciencias Básicas e Ingeniería, Universidad Autónoma Metropolitana-Iztapalapa, México City, Mexico

**Keywords:** MOFs, non-covalent interactions, gas adsorption, BioMOF, DDS, phenylethylamine derivatives, QTAIM, DFT

## Abstract

Non-covalent interactions are fundamental for understanding the chemical behavior of porous materials with guest molecules, which is key for designing new materials. The Quantum Theory of Atoms in Molecules has enabled us to visualize and analyze non-covalent interactions in host-guest systems, particularly with Metal-Organic Frameworks (MOFs) as hosts. Using this tool, we have investigated the adsorption mechanisms of highly polluting gases such as CO and ${\text{CO}}_{2}$ in InOF-1, MFM-300(Sc), and MFM-300(In). We have also explained the preferential adsorption of molecules in NOTT-401 and related changes in gas capture due to the functionalization of MOF pores. Furthermore, our group has extensively studied functionalization in the encapsulation and release of pharmacologically relevant molecules in BioMOFs, which are biocompatible, bioinspired MOFs. In this paper, we revisit our previous work while presenting new results from a systematic study of molecules from the phenylethylamine family as guests in BioMOFs, demonstrating the potential of our methodology to study new materials, such as Hydrogen-Bonded Organic Frameworks or Covalent Organic Framework.

## 1 Introduction

Research on Metal-Organic Frameworks (MOFs) has grown significantly since the late 20th century. The exceptional tunability of MOFs makes them unique materials suitable for various applications (Maleki and Taheri-Ledari, 2023), some of the most common being gas adsorption for purposes such as environmental purification, storage, or mixture separation, and drug delivery. Several articles have analyzed these applications and their theoretical studies in detail (Li et al., 2024; Peralta, 2024; Davis et al., 2025).

However, there are still challenges to overcome for the application of MOFs, such as increasing stability in water and designing structures with specific functions and properties (Khafaga et al., 2024). To address these challenges, the main avenue is the modulation of non-covalent interactions. The high efficiency of MOFs is often associated with the combined action of various interactions. It has also been shown that the stability of certain solid materials relies on the effect that hydrogen bonding networks have on the formation and electronic properties of these systems (Rajapaksha et al., 2023).

Physisorption and chemisorption are both well-established mechanisms of molecules sorption in MOFs (Sánchez-Serratos et al., 2016; Petit, 2018). In both, the nature of the metal or ligand plays a crucial role, as these factors significantly influence the intermolecular interactions between guest molecules and the MOF. Non-covalent interactions, such as hydrogen bonding, electrostatic forces, and dispersion forces, (Schneider, 2022), largely determine the interaction energy between gas molecules and the MOF.

The presence of unsaturated metal sites (UMS) (Kökçam-Demir et al., 2020) under dry conditions can promote strong physisorption or even chemisorption. While this can enhance guest uptake, it may also have undesirable effects, such as compromising the structural integrity of the MOF or hindering material recyclability.

Functionalization is an effective strategy for modulating the molecular interactions and consequently the interaction energy in MOFs (Medel et al., 2023b). This can be accomplished by modifying the ligand or altering the pore environment (Mandal et al., 2021; Eddaoudi et al., 2002). In the latter case, polar solvent molecules, such as methanol or water are commonly used. These solvent molecules primarily interact with UMS or with the inorganic metal cluster, to prevent excessive physisorption while maintaining the material’s guest uptake capacity.

Hydrogen bonds, both conventional and unconventional, are widely recognized as the primary intermolecular interactions in various chemical systems (Steiner, 2002). However, there are other types of important intermolecular interactions that require further exploration (Hobza et al., 2010; Schneider, 2022). These include dihydrogen bonds (Grabowski, 2013), H ⋅⋅⋅ H interactions (Matta, 2006), and non-covalent interactions involving heteroatoms (Alkorta et al., 2020), which can be as influential as hydrogen bonds. The strength of non-covalent interactions depends on their nature: while in strong intermolecular interactions, the electrostatic component is dominant, in weaker interactions, the dispersive component plays a more critical role. In the modeling of extended systems, periodic boundary conditions are essential; as they consistently capture the full range of intermolecular interactions. In this work, the MOFs have been studied as periodic systems, allowing for a more accurate representation of such interactions.

We have employed theoretical and computational chemistry to investigate the nature of non-covalent interactions involved in gas adsorption within MOFs. The analysis of electron density critical points provides valuable insights into the strength and nature of these interactions (Johnson et al., 2010), framed within the Quantum Theory of Atoms in Molecules (QTAIM) (Bader, 1990). The electron density, being an observable property, can be determined either experimentally or through theoretical methods. In our group, the electron density is calculated using Density Functional Theory (DFT) (Parr, 1983) within the Kohn–Sham (Kohn and Sham, 1965; Hohenberg and Kohn, 1964) framework. The subsequent analysis to identify and classify critical points is conducted using GPUAM (Graphics Processing Units for Atoms and Molecules) (Cruz et al., 2019; Hernández-Esparza et al., 2014; Hernández-Esparza et al., 2019), a specialized software developed in-house. Recently, the use of QTAIM and other electron density-based tools for describing systems dominated by intermolecular interactions has increased. However, molecular finite models predominate, which often limits the scope of the methodology and highlights the importance of using periodic calculations (Santibañez and Mendizabal, 2023). QTAIM analysis in these systems presents specific challenges, particularly because atomic basins can adopt complex geometries in crystals to accommodate ring and cage critical points de-la Roza et al. (2009). Using this methodology, we have conducted several studies on the intermolecular interactions in MOFs designed for gas trapping (Lara-García et al., 2019; Sánchez-Bautista et al., 2019; Garrido-Olvera et al., 2019; Barrios-Vargas et al., 2020; Landeros-Rivera et al., 2020; Rivera-Almazo et al., 2021). These investigations have provided a deeper understanding of the mechanisms underlying gas adsorption and the role of non-covalent interactions in these materials.

With the experience gained from studying non-covalent interactions in the adsorption of pollutant gases by MOFs, we have extended our research to another critical application of MOFs: their use as Drug Delivery Systems (DDS) (Medel et al., 2023b; Medel et al., 2023a). Drug delivery systems (DDS) refer to formulations or devices designed to distribute therapeutic substances throughout the body, improving their efficacy and helping to reduce side effects (Jain, 2008). Over the past decade, the number of publications proposing MOFs as DDSs has increased. However, some crucial features in the development of these systems remain a challenge, such as controlled drug release to avoid sudden release peaks and protection of guest molecules.

In this context, we have examined the role of pore functionalization in biocompatible MOFs (BioMOFs) (Tibbetts and Kostakis, 2020), which can be also bioinspired. In the development of these systems, in addition to biocompatibility, key features such as controlled drug release to avoid sudden bursts, and the protection of guest molecules are of utmost importance. In these characteristics, intermolecular interactions play a crucial role, influencing the stability, efficiency, and functionality of the DDS (He et al., 2021; Wang et al., 2020; Kumar et al., 2020).

In this paper, we review our contributions to two key topics: gas adsorption in MOFs and the use of BioMOFs as DDS. This review highlights the importance of characterizing non-covalent interactions in the precise design of these materials. Additionally, we emphasize the potential of theoretical and computational chemistry of periodic systems to effectively contribute to understanding the encapsulation mechanisms in MOFs and the potential it has to study other types of materials such as Hydrogen-Bonded Organic Frameworks (HOF) and Covalent Organic Framework (COFs). Furthermore, we present new theoretical results in which we propose the use of a BioMOF as a DDS for phenylethylamine derivatives.

## 2 Non-covalent interactions in MOFs

### 2.1 Gas adsorption

Environmental gases represent a major problem worldwide. Their complex composition hinders their adsorption; however, due to the tunability and adsorptive properties of MOFs, they are favorable candidates for this application (Wang et al., 2025).

In this topic, we elucidated the mechanism behind the enhancement of ${\text{CO}}_{2}$ capture through pore functionalization with methanol in InOF-1. In this case, both conventional (O-H ⋅⋅⋅ O) and unconventional (C-H ⋅⋅⋅ O) hydrogen bonds play a significant role in the interaction between the gas and the hydroxo functional group ( $\mu_{2}-$ OH) of InOF-1 (Lara-García et al., 2019). This was confirmed by experimental *in situ* DRIFTS analysis. Another study was conducted on the confinement of 2-propanol in InOF-1. However, in this case, in addition to hydrogen bonding, we observed a bottleneck effect caused by the presence of the alcohol within the pore (Sánchez-Bautista et al., 2019). A similar effect was observed when the pore was functionalized with toluene, resulting in a 1.38-fold increase in gas capture efficiency (Garrido-Olvera et al., 2019). InOF-1 pore functionalization was tested with another nonpolar solvent, small amounts of benzene were confined to investigate the adsorption of ${\text{CO}}_{2}$ and, other dangerous contaminant, ${\text{SO}}_{2}$ . The results showed a 1.6-fold increase in ${\text{CO}}_{2}$ capture; however, ${\text{SO}}_{2}$ adsorption decreased. These differences were attributed to non-covalent interactions: CO_2_ is stabilized through ${\text{CO}}_{2}\cdot \cdot \cdot \pi$ interactions; although ${\text{SO}}_{2}$ can also form such interactions, it shows stronger preference for $\mu_{2}-$ OH sites. As a result, ${\text{SO}}_{2}$ competes with benzene for preferential adsorption sites (Barrios-Vargas et al., 2020).

Building on the study of pollutant gas adsorption by InOF-1, we investigated CO capture with this MOF and identified two key interactions: $\mu_{2}-$ OH ⋅⋅⋅ O hydrogen bonds and CO ⋅⋅⋅π interactions with the phenyl rings of InOF-1 (Landeros-Rivera et al., 2020). These findings were well-supported by experimental data. Furthermore, the adsorption of CO and ${\text{SO}}_{2}$ by NOTT-401 was explored through both experimental and theoretical approaches. This MOF demonstrated exceptional stability and excellent cyclability in capturing these pollutants. Preferential adsorption sites for CO were identified using *in situ* DRIFT spectroscopy, which corresponded closely with the non-covalent interactions revealed by QTAIM analysis. Using this theoretical methodology, we also proposed the most significant interactions of ${\text{SO}}_{2}$ within NOTT-401 (Rivera-Almazo et al., 2021).

From a different perspective, some practical applications of MOFs require the activation of unsaturated metal sites (UMS), as these sites are often occupied by Lewis-Base (LB) solvent molecules. Common methods for removing LB solvent molecules and activating the UMS typically involve harsh conditions as supercritical ${\text{CO}}_{2}$ activation technique (Farha et al., 2012a; Farha et al., 2012b) and freeze-drying procedures (Ma et al., 2009). Recently, we proposed a gas flow activation technique that utilizes inert gases, such as nitrogen and argon, to displace solvent molecules from the UMS at mild temperatures (Díaz-Ramírez et al., 2025). This serves as a clear example of the importance of non-covalent interactions.

The study focused on the HKUST-1 MOF, which exhibits a specific coordination between Cu–Cu paddlewheel nodes and the oxygen atoms of the ligand. In this structure, all UMS at the Cu centers, where LB solvent molecules can bind, are oriented toward the pore, making them accessible to guest molecules. To better understand the experimental findings of the proposed technique, DFT computations and electron density analysis were conducted. The DFT results showed strong agreement with our experimental observations, further validating the approach (Díaz-Ramírez et al., 2025).

### 2.2 Drug delivery systems

The use of biomolecules as ligands for metal bonding has given rise to a new class of MOFs, known as BioMOFs, with improved biocompatibility and specific functionality (Nabipour et al., 2020). Although the biomedical use of BioMOFs is still in its early stages, reports indicate superior characteristics of BioMOFs compared to conventional bioorganic or inorganic systems (Cai et al., 2019).

**FIGURE 1:**
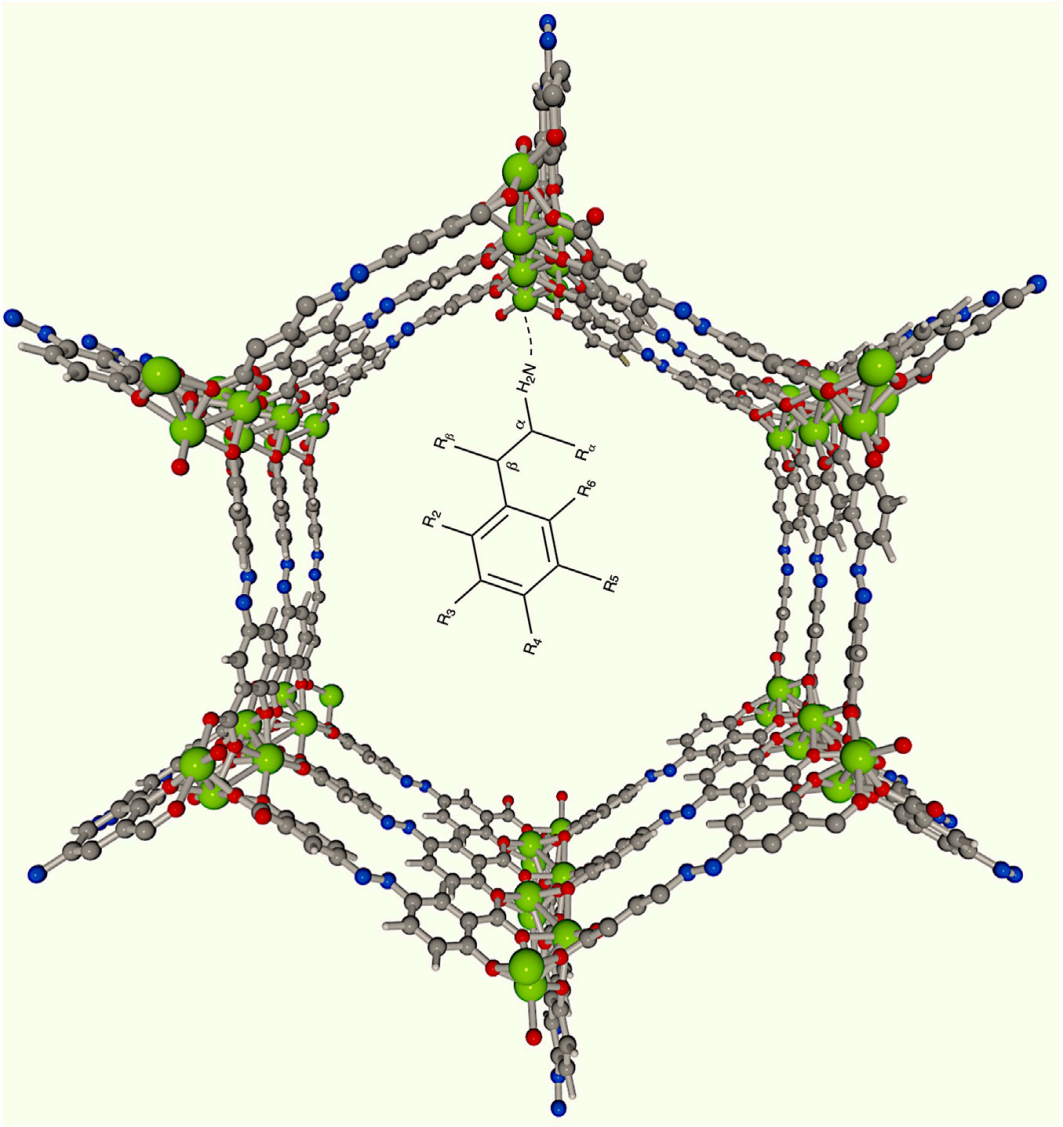
Schematic representation of the encapsulation of PHEA and its possible derivatives in a ${\text{Mg}}_{2}$ (olz) BioMOFs pore. Green represents Mg, grey C, red O, blue N and white H.

Phenylethylamine (PHEA) derivatives give rise to a wide variety of compounds related to drugs and neuroreceptors (Khan et al., 2012). Therefore, studying the encapsulation of this molecule in BioMOFs is highly relevant to advancing the understanding and design of Drug Delivery Systems (DDS). So, we investigated the encapsulation of PHEA and its derivative (Figure 1), the neuroreceptor dopamine (DA), in SU-101 BioMOFs using computational chemistry methods. Additionally, we explored the functionalization of these systems with ${\text{H}}_{2}$ O molecules. The dopamine systems were also functionalized with MeOH molecules (Medel et al., 2023b).

SU-101 BioMOFs are bioinspired MOFs that are biocompatible. They are highly stable, functionalizable, and have great potential for drug delivery, as they remain unchanged at wide pH ranges, from 2 to 14. In addition, they have been exposed to simulated physiological conditions with favorable results and have a particle size suitable for biological applications (Grape et al., 2020). BioMOF SU-101 are composed of ellagic acid as ligands, these molecules are antioxidants, and the node is formed by Bi metal, which interacts with different oxygens of the MOF structure and presents an unsaturated metal site where guest molecules may interact. Thus, our results indicate that both PHEA and DA form a Bi ⋯  N pnictogen interaction with SU-101. This is the one with the highest value of density at the bond critical point $\left(\rho_{\mathrm{BCP}}\right)$ compared to the rest of the interactions identified with QTAIM.

To study the effect of pore functionalization we started by including a water molecule with a theoretical stochastic method (García et al., 2019). This molecule formed a Bi ⋯  O interaction with the oxygen atom of the ${\text{H}}_{2}$ O molecule, among other interactions, but only with the pore walls and none with PHEA or DA. Therefore, the interaction energy $\left(E_{\mathrm{int}}\right)$ , compared to unfunctionalized systems, is not significantly modified. It was also analyzed the effect of adding four ${\text{H}}_{2}$ O molecules in the pore, located near of each UMS. In both systems, with PHEA and DA, these guest molecules are located in the center of the cavity without having access to the metal centers, and the $E_{\mathrm{int}}$ is less negative than in non-functionalized systems. Even with DA, unconventional hydrogen bonds and interactions between heteroatoms are favored by the two -OH groups in the ring. However, these interactions are weaker compared to the pnictogen ones, according to the $\rho_{\mathrm{BCP}}$ value (Tosso et al., 2020). A fifth ${\text{H}}_{2}$ O molecule, in the system with PHEA, causes two unconventional hydrogen bonds and one H ⋯  H interaction, which results in a 3 kcal change in the $E_{\mathrm{int}}$ . And with DA, a conventional hydrogen bond arises, which modifies the $E_{\mathrm{int}}$ by approximately 10 kcal.

Finally, functionalization with a MeOH molecule caused DA to shift slightly toward the UMS and realign the $\rho_{\mathrm{BCP}}$ in the pnictogen interaction; this was not observed when ${\text{H}}_{2}$ O molecules were used. Then, with four ${\text{H}}_{2}$ O molecules and one MeOH molecule, new interactions appeared with the - ${\text{CH}}_{3}$ and -OH of MeOH. The volume of MeOH molecule is larger than that of ${\text{H}}_{2}$ O and as a result, the interactions were reset. Three interactions between MeOH and DA are formed, another interaction between DA and a wall hydrogen of SU-101 appears and the $\rho_{\mathrm{BCP}}$ value for some already existing interactions changed. However, DA still presents an $E_{\mathrm{int}}$ that indicates its adsorption inside the pore is not affected. The computational results have been corroborated experimentally, using MeOH to avoid DA oxidation (Medel et al., 2023b). Thus, functionalization can modulate the $E_{\mathrm{int}}$ of the guest molecule and therefore can be applied to the design of DDS.

We explored the DDS design with ${\text{Mg}}_{2}$ (olz) BioMOFs formed by olsalazine, an anti-gastrointestinal drug, and nodes with UMS composed of Mg. These BioMOFs were first experimentally tested for the encapsulation of PHEA by Levine et al. (2016). PHEA was reported to bind to UMS via the nitrogen atom of PHEA. With this in mind, we proposed different geometries of PHEA and subsequently of DA, with the N atom pointing towards an UMS.

The most stable geometry obtained with the PHEA molecule as a guest exhibits an alkaline earth interaction, Mg ⋯  N. This is in agreement with that has been reported experimentally. This interaction has the highest percentage of contribution to $E_{\mathrm{int}}$ according to the $\rho_{\mathrm{BCP}}$ value. Subsequently, the encapsulation of the DA derivative in these BioMOFs was analyzed, obtaining similar results as PHEA. It was concluded that ${\text{Mg}}_{2}$ (olz) BioMOFs are suitable for the successful encapsulation of PHEA and its DA derivative for their application as potential drug delivery systems (Medel et al., 2023a).

### 2.3 Systematic study of phenylethylamine family

Building on the previous study, in which various PHEA and DA geometries were analyzed within ${\text{Mg}}_{2}$ (olz) pores, confirming the encapsulation of these molecules with the formation of an alkaline earth interaction, we show an example of how the methodology presented throughout this paper can be applied. Calculations were performed with various molecules of the PHEA family (phentermine, tyramine, phenylalanine, cathine, and 2C-B, dopamine was also included for comparison purposes) and the ${\text{Mg}}_{2}$ (olz) BioMOFs, using DFT at the B3LYP-D*/POB-TVPZ_rev2 theoretical level (Becke, 1993; Civalleri et al., 2008; Oliveira et al., 2019) with Crystal14 software. Details of the methodology are found in the Supplementary Material, and the results are shown in Table 1.

**TABLE 1:** Interaction energy and non-covalent interactions determined using QTAIM, Guest@Mg_2_(olz) systems. The colors of the atoms are as follows: gray C, red O, white H, blue N, cyan Br and green Mg.

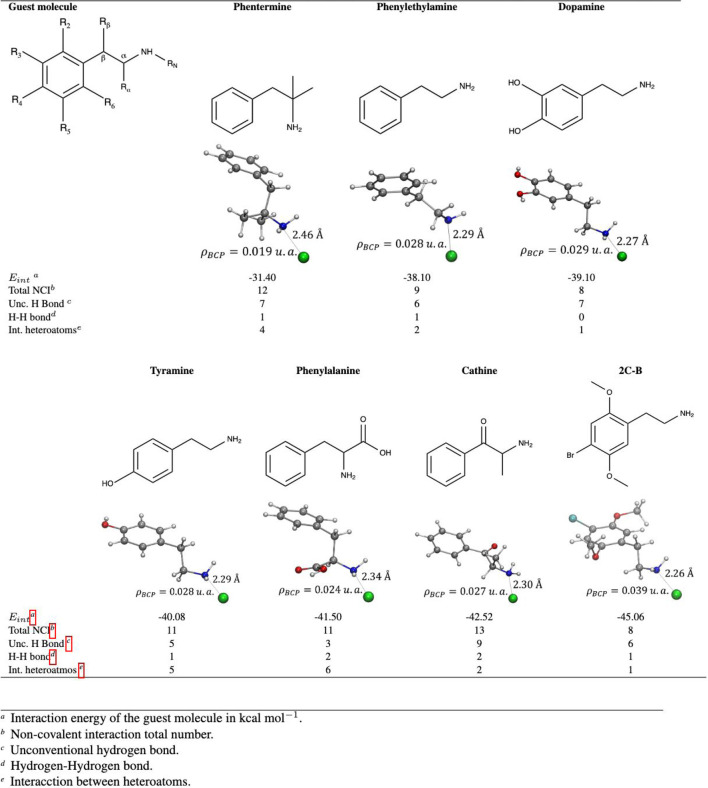

The molecules with functional groups most similar to PHEA are phentermine (PHE) and dopamine (DA). They present similar $E_{\mathrm{int}}$ , as well as the total number and types of non-covalent interactions, see Supplementary Table S1. Phentermine has only one more hydroxyl group than PHEA, and DA one more hydroxyl group than phentermine. These groups are present in the ring, in positions ${\text{R}}_{4}$ , and ${\text{R}}_{4}$ and ${\text{R}}_{3}$ respectively. In this way, the amino group is free and forms an interaction with Mg a high $\rho_{\mathrm{BCP}}$ value. This is also reflected in the Mg ⋯  N interaction distance, these data are shown in Table 1.

The $\rho_{\mathrm{BCP}}$ value in the Mg ⋯  N interaction is higher only in the case of the 2C-B molecule, which has three substituents on the ring, two epoxy and one bromine. Although Br does not form non-covalent interactions, since it is oriented towards the center of the pore, the methyls of the epoxy groups are involved in the formation of six non-covalent interactions, three unconventional hydrogen bonds, two H ⋯  H interaction and one O ⋯  O interaction. In four of the analyzed molecules, as shown in Supplementary Table S2, the amount of non-conventional hydrogen bonds explains more than 50% of the contribution to $E_{\mathrm{int}}$ according to the $\rho_{\mathrm{BCP}}$ analysis of all non-covalent interactions in each system.

On the other hand, for phenylalanine and cathine the highest percentage contribution to the $E_{\mathrm{int}}$ is represented by interactions between heteroatoms, approximately 40%. These two molecules have no substituents on the ring, but phenylalanine has a carboxylic acid at the α position and cathinone a methyl at α and a ketone at β of the carbon chain. Meanwhile, phentermine has two methyls also at the α position. The $E_{\mathrm{int}}$ of phentermine is the least negative, presenting the lowest value of the density at the critical point of binding in the Mg ⋯  N interaction. This agrees with the greater length observed between nitrogen and metal, due to the formation of six interactions between methyls and the wall of the BioMOFs. Although these are non-covalent interactions, they represent more than 40% of the contribution to the $E_{\mathrm{int}}$ , Supplementary Table S2.

Based on our analysis, it is shown that phentermine, dopamine, tyramine, cathine and 2C-B could be encapsulated in ${\text{Mg}}_{2}$ (olz), since the $E_{\mathrm{int}}$ obtained for these molecules of the PHEA family is similar to that presented by this one, and at the same time, these values are similar to DDSs successfully reported (Osorio-Toribio et al., 2020). Furthermore, the orientation of the most stable geometry found in each case coincides with that reported experimentally. Interaction energies of approximately 20–30 kcal ${\text{mol}}^{-1}$ are considered optimal for drug adsorption and delivery with slow release; our results are consistent with these values. Other factors that influence adsorption are pore size and shape, as well as functionalization (Kotzabasaki and Froudakis, 2018).

These analysis shows that molecules without substituents near the amine, such as dopamine and tyramine, present the highest contribution to the $E_{\mathrm{int}}$ by the Mg ⋯  N interaction, these can be seen in Supplementary Table S3. Likewise, the molecule 2C-B, which presents 3 substituents in the ring, is positioned closer to the node and achieves the highest $\rho_{\mathrm{BCP}}$ value for the Mg ⋯  N interaction. While for those with substituents in the carbon chain the highest percentage of contribution to the $E_{\mathrm{int}}$ is due to the formation of a greater number of interactions between heteroatoms. Finally, it is observed that the only case in which the functional groups have a drastic effect on the $E_{\mathrm{int}}$ is phentermine, since the two functional groups that characterize this molecule are located near the secondary amine. This example suggests that BioMOF ${\text{Mg}}_{2}$ (olz) serves as a DDS for molecules analogous to PHEA.

## 3 Discussion

We have shown, through theoretical and computational chemistry calculations, that it is possible to tune the interaction energy by functionalizing the pores of MOFs and BioMOFs.

The methodology we have followed, both in previous studies and in the present one, allows us to analyze the effect of pore functionalization as well as the substituent effect on the guest molecule concerning its interaction energy with the host. This methodology offers several possibilities for further exploring functionalization in the pores or the structure of MOFs. We believe that these theoretical tools and methods can contribute to the rational design of drug carriers, as well as materials for adsorbing pollutant gases.

An important pending task is including solvent effects, specially in DDS, where explicit interactions strongly influence drug release. Although challenging, finite models based on the geometries with the methodology presented here could be used with QM/MM methods or surface periodic models combined with molecular dynamics may prove useful.

From our perspective, it is possible to test the potential of a MOF or a BioMOF as a host, depending on the application, before conducting experiments. A new perspective is to test Hydrogen-bonded and Covalent Organic Framework (HOFs and COFs), such as DDS and for water treatment, respectively. In this way, the type of theoretical calculations performed in our studies can guide the design of specific materials for specific applications. HOFs are promising materials for biomedical applications due to their excellent biocompatibility, low toxicity, and high flexibility. Highly stable COFs are excelling in contaminant adsorption and heterogeneous catalysis. In HOFs, the ligands are assembled by hydrogen bonds, making the analysis of non-covalent interactions crucial to understanding these materials. While, in addition to stability, COFs present highly functionalizable ligands, which motivates us to test new horizons of functionalization.

## Data Availability

The original contributions presented in the study are included in the article/Supplementary Material, further inquiries can be directed to the corresponding author.
