# Supplementary material for: Non-covalent interactions in MOFs: a quantum approach to gas adsorption and molecular encapsulation
Source: Front Chem. 2025 Jun 6;13:1579977. doi: 10.3389/fchem.2025.1579977 (PMC12179210; doi:10.3389/fchem.2025.1579977)
Supplement: Supplementary file 1 [file DataSheet1.pdf]

# Non-Covalent Interactions in MOFs: A Quantum Approach to Gas Adsorption and Molecular Encapsulation

**Erika Medel**[1], **Rubicelia Vargas** [1,*]
[1]Departamento de Química. División de Ciencias Básicas e Ingeniería. Universidad Autónoma Metropolitana-Iztapalapa. San Rafael Atlixco 186, Col. Vicentina. Iztapalapa. AP Postal 55-534. C.P. 09340. Ciudad de México, México.
Correspondence*:
Rubicelia Vargas
rvargas@izt.uam.mx

SUPPLEMENTARY MATERIAL

## COMPUTATIONAL DETAILS

In this work the Density Functional Theory, DFT, (Parr and Weitao (1989)) was used to study periodic systems. The Crystal 14 code was used under the Kohn-Sham scheme, (Kohn and Sham (1965); Hohenberg and Kohn (1964)) with the hybrid exchange and correlation functional B3LYP, (Becke (1993)) the addition of the Grimme correction, D*, (Civalleri et al. (2008)) and the use of the POB-TVPZ_rev2 basis set (Oliveira et al. (2019)).

To select the number of points **k**, a series of single-point calculations of the structures were performed, taking the number of points **k** as a variable; with this procedure, the energy convergence was analyzed. According to this and also taking into account the space group of each system, it was decided to perform the calculations with 1 X 1 X 6 points **k** within the first Brillouin zone (David and Janice (2009)).

For the infinite series of the two-electron Coulomb and exchange integrals, a value equal to 8 was established for the parameters T1 - T4 and 20 for the last parameter, T5 (Dovesi et al. (1983); Saunders et al. (1992)).

The electron density was calculated with the Properties module of Crystal 14. With the density, the analysis of atomic connectivity was performed through the Quantum Theory of Atoms in Molecules, QTAIM (Bader (1991)).

All calculations related to QTAIM were performed using the Graphics Processing Units for Atoms and Molecules, GPUAM, code, (Hernández-Esparza et al. (2014, 2019)) in particular with the Cube3D module for periodic systems.

Functionalization was performed by stochastically adding water and methanol molecules using the Restricted Isomers Searching by Simulated Annealing, RISSA, (García et al. (2019)) code developed by the working group. The atomic positions of the host molecule of interest and the $H_2O$ or MeOH molecule, as appropriate, were then optimized.

The interaction energy $E_{int}$ was calculated with the equation,

$$E_{int} = E_{BioMOFs \cdots Guest} - E_{BioMOFs} - E_{Guest} \tag{S1}$$

where $E_{BioMOFs \cdots Guest}$ represents the energy of the total system, i.e., the BioMOFs with the molecule of interest. The $E_{BioMOFs}$ is the energy of the pristine BioMOFs, and $E_{Guest}$ is the optimization energy of the corresponding guest molecule. In the case of functionalization with $H_2O$ or MeOH, the energy of the total system corresponds to the BioMOFs with the guest molecule of interest plus the $H_2O$ or MeOH molecule(s), as applicable; and the $E_{BioMOFs}$ refers to the BioMOFs with the $H_2O$ or MeOH molecule(s).

## 1 SUPPLEMENTARY TABLES

**Table S1.** Interaction energy and non-covalent interactions determined using QTAIM. Guest@$Mg_2$(olz) systems.

| Guest molecule | $E_{int}$[a] | Total NCI [b] | Unc. H Bond [c] | H-H bond[d] | Heteroatmos int. [e] |
|---|---|---|---|---|---|
| **Phentermine** | -31.40 | 12 | 7 | 1 | 4 |
| **Phenylethylamine** | -38.10 | 9 | 6 | 1 | 2 |
| **Dopamine** | -39.10 | 8 | 7 | 0 | 1 |
| **Tyramine** | -40.08 | 8 | 6 | 1 | 1 |
| **Phenylalanine** | -41.50 | 11 | 5 | 1 | 5 |
| **Cathine** | -42.52 | 11 | 3 | 2 | 6 |
| **2C-B** | -45.06 | 13 | 9 | 2 | 2 |

[a] Interaction energy of the guest molecule in kcal $mol^{-1}$.
[b] Non-covalent interaction total number.
[c] Unconventional hydrogen bond.
[d] Hydrogen-Hydrogen bond.
[e] Heteroatomos interacction.

**Table S2.** Percentage contribution (%) to interaction energy by type of non-covalent interactions in each system Guest@$Mg_2$(olz).

| Types NCI[a] | Phentermine | Phenylethylamine | Dopamine | Tyramine | Phenylalanine | Cathine | 2C-B |
|---|---|---|---|---|---|---|---|
| **Mg···N** | 18.8 | 38.3 | 39.8 | 40.0 | 25.3 | 27.0 | 25.7 |
| **Heteroatmos int.** [b] | 30.8 | 8.8 | - | - | 41.0 | 40.5 | 4.2 |
| **Unc. H Bond** [c] | 47.8 | 51.5 | 60.2 | 57.2 | 30.0 | 21.1 | 54.0 |

[a] Types of Non-covalent interaction.
[b] Heteroatomos interactions.
[c] Unconventional hydrogen bond.

**Table S3.** Electronic density and properties of this in the bond critical point for the interaction Mg···N using QTAIM for Guest@$Mg_2$(olz) systems. The length of the interaction is also shown.

| Guest molecule | $\rho_{BCP}$ [a] | $\nabla^2_{BCP}$ [b] | G[c] | V [d] | H [e] | Int. length [f] |
|---|---|---|---|---|---|---|
| **Phentermine** | 0.019 | 0.098 | 0.02 | -0.016 | 0.004 | 2.46 |
| **Phenylethylamine** | 0.028 | 0.148 | 0.032 | -0.027 | 0.005 | 2.29 |
| **Dopamine** | 0.029 | 0.154 | 0.034 | -0.029 | 0.005 | 2.27 |
| **Tyramine** | 0.028 | 0.169 | 0.036 | -0.029 | 0.007 | 2.29 |
| **Phenylalanine** | 0.024 | 0.161 | 0.033 | -0.025 | 0.008 | 2.34 |
| **Cathine** | 0.027 | 0.171 | 0.036 | -0.028 | 0.007 | 2.3 |
| **2C-B** | 0.039 | 0.277 | 0.059 | -0.049 | 0.01 | 2.26 |

[a] Electronic density at the bond critical point.
[b] Laplacian of ectronic density at the bond critical point.
[c] Kinetic energy at the bond critical point.
[d] Potential energy at the bond critical point.
[e] Total electronic density energy at the bond critical point.
[f] Interaction length in angstrom (Å).

## REFERENCES


Bader, R. F. (1991). A quantum theory of molecular structure and its applications. *Chemical Reviews* 91, 893–928. doi:10.1021/cr00005a013

Becke, A. D. (1993). A new mixing of Hartree-Fock and local density-functional theories. *The Journal of Chemical Physics* 98, 1372–1377. doi:10.1063/1.464304

Civalleri, B., Zicovich-Wilson, C. M., Valenzano, L., and Ugliengo, P. (2008). B3LYP augmented with an empirical dispersion term (B3LYP-D*) as applied to molecular crystals. *CrystEngComm* 10, 405–410. doi:10.1039/b715018k

David, S. S. and Janice, A. S. (2009). *Density Functional Theory, A Practical Introduction* (John Wiley & Sons)

Dovesi, R., Pisani, C., Roetti, C., and Saunders, V. R. (1983). Treatment of coulomb interactions in hartree-fock calculations of periodic systems. *PHYSICAL REVIEW B* 28, 5781–5792

García, J. J., Hernández-Esparza, R., Vargas, R., Tiznado, W., and Garza, J. (2019). Formation of small clusters of NaCl dihydrate in the gas phase. *New Journal of Chemistry* 43, 4342–4348. doi:10.1039/c8nj06315j

Hernández-Esparza, R., Mejía-Chica, S. M., Zapata-Escobar, A. D., Guevara-García, A., Martínez-Melchor, A., Hernández-Pérez, J. M., et al. (2014). Grid-based algorithm to search critical points, in the electron density, accelerated by graphics processing units. *Journal of Computational Chemistry* 35, 2272–2278. doi:10.1002/jcc.23752

Hernández-Esparza, R., Álvaro Vázquez-Mayagoitia, Soriano-Agueda, L. A., Vargas, R., and Garza, J. (2019). GPUs as boosters to analyze scalar and vector fields in quantum chemistry. *International Journal of Quantum Chemistry* 119, e25671. doi:10.1002/qua.25671

Hohenberg, P. and Kohn, W. (1964). Inhomogeneous electron gas. *Physical Review* 136, B864. doi:10.1103/PhysRev.136.B864

Kohn, W. and Sham, L. J. (1965). Self-cosistent equations including exchange and correlation effects. *Physical review* 140, A1133–A1138

Oliveira, D. V., Laun, J., Peintinger, M. F., and Bredow, T. (2019). Bsse-correction scheme for consistent gaussian basis sets of double- and triple-zeta valence with polarization quality for solid-state calculations. *Journal of Computational Chemistry* 40, 2364–2376. doi:10.1002/jcc.26013

Parr, R. G. and Weitao, Y. (1989). *Density functional theory of atoms and molecules*

Saunders, V. R., Freyria-Fava, C., Dovesi, R., Salasco, L., and Roetti, C. (1992). On the electrostatic potential in crystalline systems where the charge density is expanded in gaussian functions. *Molecular Physics* 77, 629–665. doi:10.1080/00268979200102671
